# An Adaptive Routing Algorithm Based on Relation Tree in DTN

**DOI:** 10.3390/s21237847

**Published:** 2021-11-25

**Authors:** Diyue Chen, Hongyan Cui, Roy E. Welsch

**Affiliations:** 1 State Key Laboratory of Networking and Switching Technology, Beijing University of Posts and Telecommunications, Beijing 100876, China; chendiyue@bupt.edu.cn; 2 Beijing Laboratory of Advanced Information Networks, Beijing University of Posts and Telecommunications, Beijing 100876, China; 3 Sloan School of Management and Center for Statistics and Data Science, Massachusetts Institute of Technology, Cambridge, MA 02139, USA; rwelsch@mit.edu

**Keywords:** DTN, routing algorithm, relation tree, adaptive

## Abstract

It is found that nodes in Delay Tolerant Networks (DTN) exhibit stable social attributes similar to those of people. In this paper, an adaptive routing algorithm based on Relation Tree (AR-RT) for DTN is proposed. Each node constructs its own Relation Tree based on the historical encounter frequency, and will adopt different forwarding strategies based on the Relation Tree in the forwarding phase, so as to achieve more targeted forwarding. To further improve the scalability of the algorithm, the source node dynamically controls the initial maximum number of message copies according to its own cache occupancy, which enables the node to make negative feedback to network environment changes. Simulation results show that the AR-RT algorithm proposed in this paper has significant advantages over existing routing algorithms in terms of average delay, average hop count, and message delivery rate.

## 1. Introduction

Traditional Internet networks are based on the TCP/IP protocol and rely on physical links that need to meet the characteristics of bidirectional, end-to-end continuous stability. However, as the research field continues to expand, more diverse and complex network environments have emerged, such as the interstellar Internet [1], social networks [2], vehicle-mounted networks [3,4], wireless sensor networks [5], and so on. In these networks, even if end-to-end transmission paths exist, they are relatively susceptible to interruptions, small data transfer rates, and long transmission delays. In these application scenarios, many serious challenges are encountered when using the traditional TCP/IP protocol for network communication.

To solve the data transmission problem in these networks, Kevin Fall first proposed the Delay Tolerant Network (DTN) model in 2003 [6]. When a message arrives at a node, that node will first store the message and carry it through the network. When the node carrying the message needs to pass the message to other nodes, the message will be moved from a storage location on that node to a storage location on another node. In this way, the message is transmitted until the delivery of the message is completed or the message lifecycle is exhausted and discarded. The “carry-store-forward” model overcomes the problems associated with intermittent connections, long delays, variable network topologies, and high bit error rates, improving the quality of network communications [7]. The research of DTN provides strong scientific theory and technical support for message interaction in military warfare, aerospace communication, disaster recovery, emergency rescue, and other fields.

In DTN, nodes may be in constant motion, the network does not have a fixed topology, and there is usually no end-to-end network connection between nodes, so a series of routing algorithms based on stable topology in traditional Internet are not applicable to DTN, which makes routing algorithms one of the most important research areas in DTN [8,9]. Nodes in opportunistic networks often refer to vehicles or users carrying wireless short-range communication devices, and these nodes participating in social scenarios also have unique social attributes [10], so applying social attributes in DTN routing algorithms has attracted a great deal of attention from researchers in recent years.

In this paper, we propose an adaptive routing algorithm based on Relation Tree (AR-RT) to address the above issues, and the main contributions are summarized as follows:

- We propose a new method for constructing the Relation Trees, which is based on the fact that in most application scenarios of DTN, nodes exhibit social attributes.
- We propose a sub-policy forwarding method based on the Relation Tree, which is capable of more targeted forwarding based on the social attributes of each node.
- We propose an adaptive replication method based on node cache occupancy that can adapt to the dynamically changing network environment.

The rest of the paper is organized as follows. We discuss related work in Section 2, Section 3 provides a brief overview of the AR-RT algorithm, Section 4 describes how to construct the Relation Tree, Section 5 describes the adaptive sub-policy replication forwarding approach of the AR-RT algorithm, and in Section 6, we demonstrate the performance of the AR-RT algorithm based on four key parameters by comparing it with existing algorithms. In Section 7, we conclude the whole paper.

## 2. Related Work

The routing problem is one of the hot issues in DTN research. DTN routing algorithms can be divided into two types, zero-information type and auxiliary information type, depending on whether external auxiliary information is required [11]. Direct delivery [12] is the simplest zero-information routing algorithm, where the source node generates a message and will always carry it in the network and will not forward it additionally. This algorithm has the lowest network overhead due to the absence of redundant relay operations, but its performance in terms of transmission efficiency and the average delay is poor. The Epidemic routing algorithm [13] simulates the propagation of an infectious virus in a biological environment by forwarding the message to each node it encounters. However, with limited resources, the nature of blind message propagation will lead to a buffer overflow, and as the number of copies in the network increases, a large number of copies take up network resources and can easily cause network congestion. To overcome the problems caused by the Epidemic routing algorithm, the Spray-and-Wait routing algorithm [14] was proposed, which is divided into two main phases: spray and wait. In the spray phase, the source node copies the maximum number of message copies, which is fixed, and forwards a copy of the message every time it encounters an intermediate node until only one copy of the message remains. If the node carries only a copy of the message, it will enter the waiting phase, in which the message is forwarded only when the target node is encountered. The Binary Spray-and-Wait algorithm [15] is an improvement of the original Spray-and-Wait algorithm. In the spray phase, half forwarding is used to effectively improve the message delivery rate. The maximum message copy number, L, is the key parameter that determines the performance of the Spray-and-Wait algorithm. The paper [16] controls L by controlling message regeneration and deletion. This method extends the application of the algorithm by changing the value of the maximum message copy number, L, from a fixed value to a variable value that can be adapted to the application environment. However, the above zero-information routing algorithms do not consider the differences between nodes [17]. They cannot develop a reasonable routing strategy based on the differences, resulting in inefficient transmission.

The external auxiliary information includes historical encounter probability, geographic location, etc. This external auxiliary information plays a huge role in improving forwarding efficiency. Prophet [18] is a probability-based routing algorithm that uses historical transmission probabilities to calculate the current transmission probability. The node carrying the message will decide whether it should forward by comparing the expected probability of encountering the destination node. In order to improve the delivery rate of IoT applications in DTN, the authors of [19] propose a scheduling-probability routing algorithm based on node encounter history and convertibility, using two scheduling mechanisms to extend the traditional Prophet algorithm. The authors of [20] proposed the Sharing Spray-and-Wait algorithm. This algorithm uses the Markov chain network model to analyze the message carrying time and delivery predictability, and then uses the results to select the next node and the number of message copies to be delivered in the next hop. The authors of [21] enhanced the Spray-and-Wait algorithm by changing the number of copies sent from the sender to the receiver. The authors of [22] proposed the Geography-Based Adaptive Spray (GBAS) routing algorithm to improve the Spray-and-Wait routing algorithm in many aspects, mainly by calculating the activity range of the destination node in the spray phase to select the appropriate next-hop node. 

Considering that the social attributes of nodes are stable over time, researchers have proposed many social-based routing algorithms. The Bubble Rap algorithm [23] is a representative community-division-based routing algorithm. The algorithm uses the encounter law between nodes to divide the communities, and designs two different mechanisms for forwarding messages within and between communities. The authors of [24] proposed a technique to detect the selfishness of nodes based on their historical message forwarding and discarding behaviors, and a novel credit-based scheme to motivate nodes to cooperate in forwarding messages. The authors of [25] extracted the key factors affecting friendships, including contact frequency, contact time, and regularity of contact through the analysis of social networks, and friendships in turn include direct friendships and indirect friendships, thus constructing an analytical model to evaluate the closeness of friendships. The authors of [26] predicted the mobile probability based on the social relationships and forwarding partnerships among mobile users, using a hybrid relationship matrix decomposition to predict the mobile encounter probability of users. The authors of [27] introduced a new metric, social energy, to quantify the ability of a node to forward packets to others. Social energy is generated through the encounter of nodes, shared by the community of encountering nodes, and decays over time. The authors of [28] designed a novel opportunity network mobility model for node community hierarchy to deeply evaluate the similarity and difference of nodes at different levels in the community and to implement relay node selection and destination node difference analysis in different community hierarchies. The authors of [29] combined location-based forwarding with contact-based forwarding, relying on packets based on predictable location and contact patterns.

The various auxiliary information types of algorithms described above require each node to maintain real-time information from other nodes, so when the network size increases, the nodes lack the adaptive adjustment capability to reduce this part of the overhead.

## 3. Overview

In this section, we propose an adaptive routing algorithm based on the Relation Tree (AR-RT) in DTN. Besides, the summary of important used variables in the paper is listed in Table 1.

**Figure 1:**
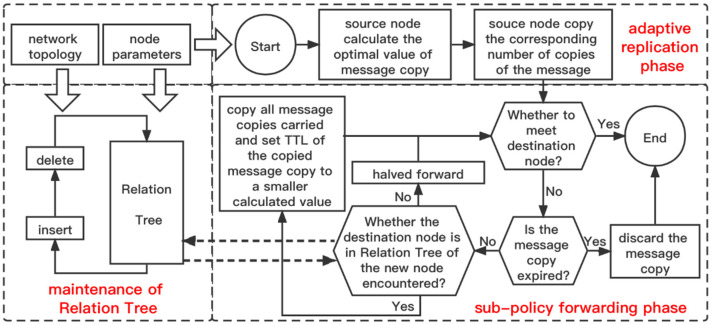
The flowchart of the AR-RT algorithm.

All nodes in the network topology maintain a Relation Tree with themselves as the root node and update the Relation Tree in real time. The AR-RT algorithm is divided into two phases: the adaptive replication phase, and the sub-policy forwarding phase. Whenever a source node initiates a forwarding task, it first enters the adaptive replication phase, in which it replicates the most appropriate number of message copies based on its own cache occupancy. From the time the source node encounters the first relay node that is not the destination node, it randomly enters the sub-policy forwarding phase, in which the node carrying the message adopts a different forwarding strategy depending on whether the Relation Tree of the encountered relay node contains the destination node of the message, and all subsequent relay nodes adopt the same forwarding strategy until they encounter the destination node to complete the final forwarding of the message. The entire flowchart is shown in Figure 1.

## 4. Relation Tree

Introducing the concept of social networks into routing algorithms is the latest trend in the field of DTN research. In many application scenarios of DTNs, the nodes are mostly mobile devices with communication functions used by people, and thus the nodes in DTNs also exhibit social characteristics similar to those of humans. Despite the relatively unpredictable network topology, human movement patterns are not completely random; for example, closely related nodes have a higher probability of meeting, and these nodes that meet frequently form a social circle. Compared with nodes outside the social circle, nodes within the same social circle are more closely connected, meet more frequently, and have a higher probability of successfully delivering messages to each other. If we can accurately construct the social circles of every node, we can forward messages in a more targeted manner and improve the forwarding efficiency. Based on the above analysis, we propose a method for constructing social circles, which is called a Relation Tree because it is reflected in the storage structure as a tree storage structure.

Definition (Relation Tree (RT)). Each node in the network maintains a Relation Tree with itself as the root node. the maximum depth of the Relation Tree is 3, the memory cycle of each node is T, and the judgment threshold of the number of encounters is M. A node with depth 2 in the Relation Tree satisfies the condition that the number of encounters with the root node exceeds M in a memory cycle, and a node with depth 3 in the Relation Tree satisfies the condition that the number of encounters with its parent node exceeds M in a memory cycle.

### 4.1. Maintenance of the Relation Tree

At the end of each cycle, the Relation Tree is updated in real-time, and the main operations are insertion and deletion. The process of updating the Relation Tree is shown in Algorithm 1.
**Algorithm1.** Updating Relation Tree**Input:**    $V=\{v_{i}|1<i<n\}$, set of nodes in the network $N(v_{i},v_{j}|v_{i}\in V,v_{j}\in V)$, the number of times $v_{i}$ and $v_{j}$ meet in a memory cycle T $RT_{i}$, the relation tree of node $v_{i}$
 $RT_{j}$, the relation tree of node $v_{j}$
 M, relationship threshold 1:   **if**   $v_{i}$ encounters $v_{j}$ and $N\left(v_{i},v_{j}\right)=M-1$  **then** 2:       add $v_{j}$ as a child node of $v_{i}$ in $RT_{i}$
3:       **for**   each child node $c_{j}$ of $v_{j}$ in $RT_{j}$  **do** 4:        **if**   $c_{j}\notin RT_{i}$   **then** 5:         add $c_{j}$ as a child node of $v_{j}$ in $RT_{i}$
6:        **end if** 7:       **end for** 8:   **end if** 9:   **for**   each child node $c_{i}$ of $v_{i}$ in $RT_{i}$   **do** 10:       **if**   $N\left(c_{i},v_{i}\right)<M$   **then** 11:        remove $c_{i}$ from $RT_{i}$
12:       **end if** 13:   **end for**

#### 4.1.1. Tree Insertion

Whenever the root node A encounters a new node B so that the number of encounters between them in a memory cycle reaches M, an insertion operation is performed on the Relation Tree of A. The insertion operation includes:

1. Add the new node B to become a child of the root node A.
2. Add all nodes in the original Relation Tree of B that are not nodes in the Relation Tree of A and whose encounter times with B exceeds M (nodes in the Relation Tree of B with depth 2 and not in the Relation Tree of A) to become children node of B in the Relation Tree of A.

**Figure 2:**
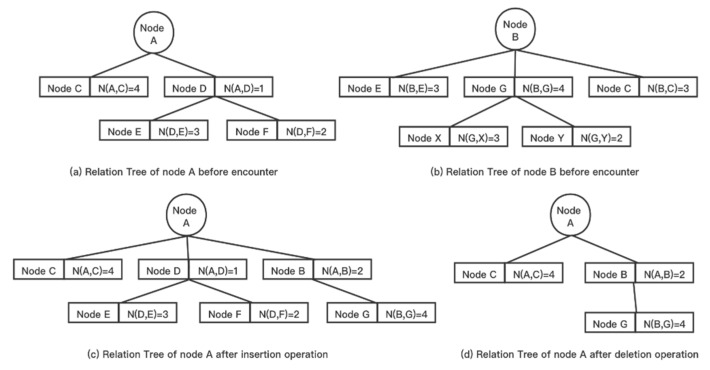
Maintenance of the Relation Tree.

If M = 2, when node A and node B meet again in the same memory cycle, the number of times they meet reaches the judgment threshold, as shown in Figure 2. Since nodes C and E with depth 2 in the Relation Tree of B are already in the Relation Tree of A, only node G is added to become a child node of B in the Relation Tree of A.

#### 4.1.2. Tree Deletion

At the end of each cycle, all nodes of the Relation Tree whose number of encounters is below M with depth 2 and their children are deleted. If M = 2, node D and all its child nodes that are less than 2 are deleted at the end of the cycle, as shown in Figure 2.

### 4.2. Important Parameters Related to the Relation Tree

In our algorithm, the nodes with depth 2 represent the “friends” of the root node, and the nodes with depth 3 represent the “friends of friends” of the root node. The Relation Tree is updated in real-time. The size of the Relation Tree depends on the memory cycle T and the judgment threshold M. The optimal value of these two depends on the number of nodes, the movement speed of nodes, the storage space of nodes, etc. The user can adjust the size of the Relation Tree according to the actual parameters of the network.

## 5. Algorithm Design

The AR-RT algorithm is divided into two phases: adaptive replication phase and sub-policy forwarding phase.

### 5.1. Adaptive Replication Phase

Whenever a source node initiates a forwarding task, it enters that phase first. In this phase, the most appropriate number of message copies is copied based on its own cache occupancy. The messages that occupy the node cache are mainly divided into two categories: one is the newly generated messages by the node and the other is the messages received by the node as a relay node. When a message expires, the node automatically discards the message, so expired messages are not included in the above two categories. The cache of each node in the network has an initial size: buffer size. The calculation formula of node cache occupancy rate, $\beta_{now},$ is as in Equation (1):

(1) $$\beta_{now\;}=\frac{\sum_{i=1}^{X}Received\left\{m_{i}\right\}+\sum_{i=1}^{Y}Created\left\{m_{i}\right\}}{Buffersize}$$

where *X* represents the total number of messages successfully received by this node before the current time, and *Y* represents the total number of messages successfully created by this node before the current time.

When the source node starts to send data to other nodes, it will copy L copies of the message. In the AR-RT algorithm, the source node can dynamically adjust L according to its own buffer occupancy. The calculation formula of L is as in Equation (2):

(2) $$L=L_{init}-\partial *\left(\beta_{now\;}-\beta_{best}\right)$$

where $L_{init}$ is an initial set value,$\;\beta_{best}$ is the optimal buffer occupancy rate of all nodes that are initially set, and ∂ is an adjustment factor. This phase provides negative feedback that helps maintain the overall network load level at an appropriate level.

### 5.2. Sub-Policy Forwarding Phase

As shown in Algorithm 2, when a node carrying copies of a message encounters a new node, it adopts a different forwarding strategy until it reaches the destination node, depending on whether the new node contains the destination node in its Relation Tree:

1. If the Relation Tree of the new node does not contain the destination node, half of the message copies carried by themselves (rounded down) are forwarded to the new node. This forwarding strategy uses halving propagation to improve the efficiency of breadth-first forwarding among unfamiliar nodes.
2. If the Relation Tree of the new node contains the destination node, all the message copies carried are copied and forwarded to the new node, and the survival time ($TTL_{new}$) of these newly copied generated message copies is shown in Equation (3). Where M<T, ensuring $TTL_{new}$ < $TTL_{old}$, because the destination node is in the Relation Tree of the new node, the encounter expectation is higher, and the message copies generated by the new replication only need a smaller survival time to avoid wasting network resources. This forwarding strategy can effectively accelerate the forwarding efficiency in the final stage of messages by exploiting the flooding effect while keeping the negative impact of the flooding effect in a localized range within a short period of time.

(3) $$TTL_{new}=TTL_{old}*\;\frac{M}{T}$$

The purpose of using different forwarding policies is to perform more targeted forwarding based on the social attributes of each node to reduce the impact of flooding and reduce network overhead while improving forwarding efficiency and reducing delay.

**Algorithm2.** Forwarding Phase**Input:** $V=\{v_{i}|1<i<n\}$, set of nodes in the network $N(v_{i},v_{j}|v_{i}\in V,v_{j}\in V)$, the number of times $v_{i}$ and $v_{j}$ meet in a memory cycle T $RT_{i}$, the relation tree of node $v_{i}$
 $RT_{j}$, the relation tree of node $v_{j}$
 M, relationship threshold T, memory cycle $L_{i}\left(m_{a}\right)$, the number of copies of $m_{a}$ carried by node $v_{i}$
1:   **if**   $v_{i}$ encounters $v_{j}$ **then** 2:    **for   ** each message $m_{a}$ in $v_{i}$   **do** 3:     **if**   $m_{a}.TTL>0$   **then** 4:      $v_{d}=m_{a}^{\prime }$ destination 5:      **if**   $v_{j}==v_{d}$   **then** 6:       $v_{i}$ directly forwards $m_{a}$ to $v_{j}$
7:      **else if**   $v_{d}\in RT_{j}$   **then** 8:       $L_{j}\left(m_{a}\right)=L_{i}\left(m_{a}\right)$
9:       $v_{j}.m_{a}.TTL=v_{j}.m_{a}.TTL*M/T$
10:      **else then** 11:       $L_{j}\left(m_{a}\right)=\lfloor L_{i}\left(m_{a}\right)/2\rfloor$
12:       $L_{i}\left(m_{a}\right)=\lceil L_{i}\left(m_{a}\right)/2\rceil$
13:      **end if**
14:     **end if** 15:    **end for** 16:   **end if**

## 6. Simulation

In this section, we use ONE simulation software [30] to test the performance of each algorithm. The algorithms we selected for comparison are Epidemic, Spray-and-Wait (L = 8), Prophet ($P_{init}$ = 0.75, *β* = 0.25, and *γ* = 0.98), and Bubble Rap. In order to better evaluate the performance of routing algorithms, this paper mainly uses four indicators: delivery rate, average delay, overhead, and average hop count.

### 6.1. Simulation Scenarios and Parameters

For a more objective comparison with Spray-and-Wait, we also set the initial number of message copies, $L_{init}$, to 8. Based on $L_{init}$ = 8, we found suitable values for the other parameters through several tests. All specific parameters are shown in Table 2. In this section, the simulation is performed for Vehicle Ad Hoc Network (VANET), a self-organizing network, and the map used is the city map of Helsinki, with a total of four groups of nodes with different attributes, and the attributes of each group of nodes are shown in Table 3. The nodes in group 1 and group 3 will be randomly assigned their points of interest on the map. The probability of nodes selecting their corresponding points of interest as new destination locations is higher than that of other locations on the map. After determining the destination location, the node will first calculate the shortest path based on the road and then follow the road to the destination. The probability of selecting different points of interest as the new destination location is also random and different. The nodes in group 2 and group 4 follow their respective fixed lines of movement. This is to simulate the different working attributes of different network nodes in the real DTN scene.

### 6.2. Simulation Results

By varying the number of nodes, message TTL value, message generation interval, and node cache size, we can analyze the performance of the five algorithms in different scenarios. In order to make the simulation results more accurate, each simulation scenario was repeated several times with different random seeds. 

#### 6.2.1. Impact of The Number of Nodes

**Figure 3:**
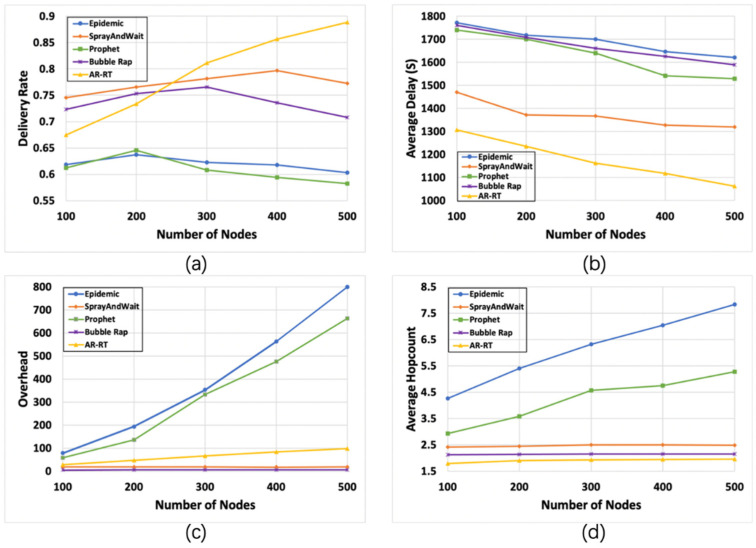
Comparison of the results (95% CI) of various algorithms under different numbers of nodes. Subfigures (**a**–**d**) compare the performance of the five algorithms with the variation of the four parameters, delivery rate (**a**), average delay (**b**), overhead (**c**) and average hop count (**d**), with the number of nodes, respectively.

The results for different numbers of nodes are shown in Figure 3. Figure 3a shows that when increasing the number of nodes, the delivery rate of AR-RT increases significantly, while that of the other algorithms does not fluctuate much, which is because the higher the number of nodes, the more accurate AR-RT is in determining the social attributes among the nodes. It is worth noting that after the number of nodes exceeds 300, the delivery rate of Bubble Rap starts to decrease due to the increase in complexity of calculating the node centrality with the number of nodes, resulting in inaccurate results. Figure 3b shows that as the number of nodes increases, the average delay of all four algorithms decreases, with AR-RT having the smallest average delay, which is due to the fact that AR-RT is more efficient in forwarding the propagation phase in the final Relation Tree, reducing the overall delay. Figure 3c shows that the network overhead of Epidemic and Prophet is increasing significantly as the number of nodes increases, due to the uncapped replication of these two algorithms, and the network overhead of these two algorithms in a high node density scenario can have a serious impact on the network. The network overhead of AR-RT increases slightly but is within an acceptable range, and the network overhead of Bubble Rap remains stable and is always at the lowest. Figure 3d shows that the average hop count of Epidemic and Prophet increases significantly as the number of nodes increases, reflecting the inefficient forwarding of these two algorithms. The average hop counts of the remaining three algorithms remain basically stable, among which AR-RT performs better due to the high forwarding efficiency caused by using different forwarding methods based on the Relation Tree.

#### 6.2.2. Impact of TTL

**Figure 4:**
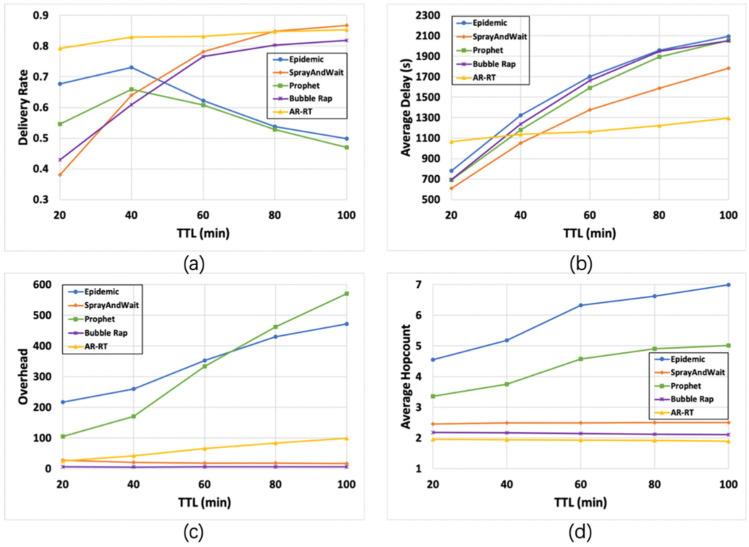
Comparison of the results (95% CI) of various algorithms under different TTL. Subfigures (**a**–**d**) compare the performance of the five algorithms with the variation of the four parameters, delivery rate (**a**), average delay (**b**), overhead (**c**) and average hop count (**d**), with TTL, respectively.

Figure 4 shows the impact of TTL. Figure 4a shows that the delivery rate of Spray-and-Wait and Bubble Rap is low in the scenario of low TTL, and with the increase TTL, the delivery rate increases rapidly and eventually tends to be high, which reflects the poor adaptability of these two algorithms to different attributes of the service. The delivery rate of the AR-RT algorithm remains stable and always at a high level, and Epidemic and Prophet both increase and then decrease, and the overall delivery rate is low. Figure 4b shows that the average delay of AR-RT increases slightly with the increase of TTL, and the advantage is obvious in the high TTL scenario. The increasing trend of the remaining four algorithms is obvious. Figure 4c shows that the network overhead of Epidemic and Prophet is increasing significantly as TTL increases, the network overhead of AR-RT increases slightly but is within an acceptable range, and the network overhead of Bubble Rap remains basically stable and is always in the lowest state. Figure 4d shows that with the increase of TTL, the average hop count of Epidemic and Prophet increases significantly and is at a higher level, reflecting the inefficiency of forwarding of these two algorithms. The average hop count of the remaining three algorithms remains basically stable, with AR-RT performing better.

#### 6.2.3. Impact of Message Generation Interval

**Figure 5:**
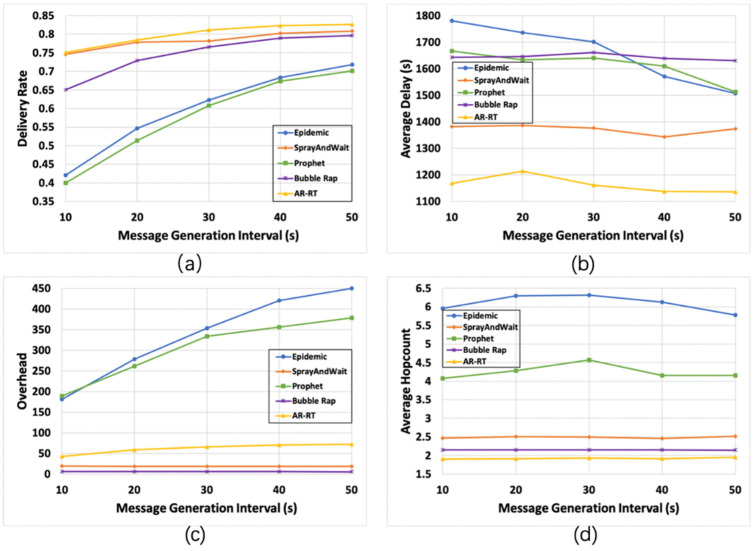
Comparison of the results (95% CI) of various algorithms under different message generation intervals. Subfigures (**a**–**d**) compare the performance of the five algorithms with the variation of the four parameters, delivery rate (**a**), average delay (**b**), overhead (**c**) and average hop count (**d**), with the message generation interval, respectively.

Figure 5 shows the impact of the message generation interval. From Figure 5a, we can see that the delivery rates of Epidemic and Prophet are very low when the increase of the message generation interval is small, which indicates that in the high-intensity transmission scenario, the high message discard rate caused by the uncapped replication method will seriously reduce the final delivery rate of messages. In the low-intensity transmission scenario, the delivery rate of AR-RT has an advantage due to its ability to dynamically adjust the number of copies of the initial message. From Figure 5b, it can be seen that the average delay of Epidemic and Prophet gradually decreases as the message generation interval increases, but always remains at a high level. The average delay of Bubble Rap remains stable and at a high level, and the average delay of the remaining two algorithms remains stable and at a low level, with the average delay of AR-RT being smaller. Figure 5c shows that the network overhead of Epidemic increases significantly as the message generation interval increases, the network overhead of Prophet increases and then stabilizes, the network overhead of AR-RT increases slightly but is within an acceptable range, and the network overhead of Bubble Rap remains stable and is always at the lowest level. Figure 5d shows that as the message generation interval increases, the average hop count of Epidemic and Prophet increases and then decreases, but always remains at a high level, and the average hop count of the remaining three algorithms remains stable, with AR-RT performing better.

#### 6.2.4. Impact of Buffer Size

**Figure 6:**
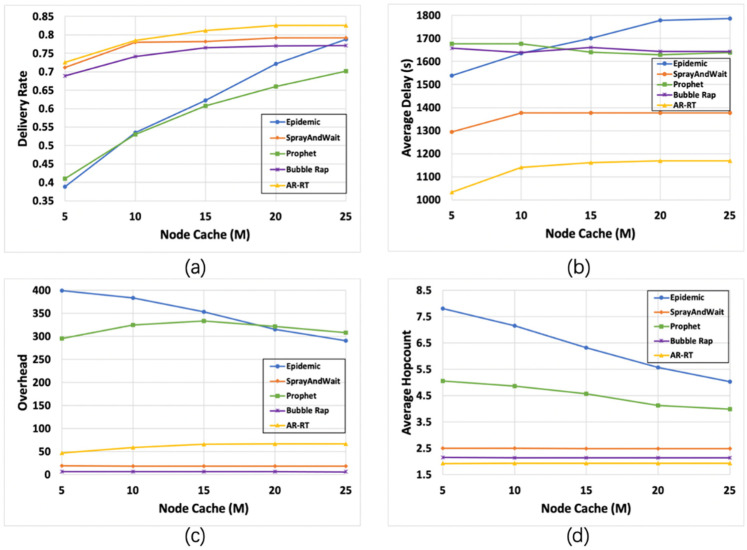
Comparison of the results (95% CI) of various algorithms under different node caches. Subfigures (**a**–**d**) compare the performance of the five algorithms with the variation of the four parameters, delivery rate (**a**), average delay (**b**), overhead (**c**) and average hop count (**d**), with the node cache, respectively.

The results for different buffer sizes are shown in Figure 6. From Figure 6a, we can see that the delivery rate of Epidemic and Prophet is low when the buffer size is small, because the high message discard rate brought by the uncapped replication method will seriously reduce the final delivery rate of messages. The delivery rate of the remaining three algorithms remains stable and at a high level, and when the buffer size exceeds 15M, AR-RT has an advantage due to its ability to dynamically adjust the number of copies of the initial message. Figure 6b shows that as the buffer size increases, the average delay of Epidemic increases slightly, while the average delay of the remaining four algorithms remains stable, with AR-RT performing the best and having a clear advantage. Figure 6c shows that the network overhead of Epidemic tends to decrease as the buffer size increases. The network overhead of the remaining four algorithms remains basically stable, with the network overhead of Bubble Rap being in the lowest state. Figure 6d shows that as the buffer size increases, the average hop count of Epidemic and Prophet shows a significant decreasing trend, but always at a high level, and the average hop count of the remaining three algorithms remains stable, among which AR-RT performs better.

## 7. Summary

In this paper, we proposed an adaptive routing algorithm based on the Relation Tree in DTN. We exploited the feature that nodes in DTN have stable social attributes similar to people and introduced the concept of the Relation Tree. Each node constructs its own Relation Tree based on its historical encounter frequency, and will adopt different forwarding policies based on the Relation Tree in the forwarding phase. In addition, the source node adaptively adjusts the initial maximum number of message copies according to its own cache occupancy, which enables the node to make negative feedback to the network environment changes. Simulation results show that the proposed AR-RT algorithm had the best overall performance in terms of average delay, average hop count, and delivery rate compared with existing routing algorithms, while the network overhead rate was kept at a stable low level.

## Figures and Tables

**Table 1:** List of notations.

Notation	Explanation
T	Memory cycle
M	Relationship threshold
V	Set of nodes in the network
$N\left(v_{i},v_{j}\right)$	Number of times $v_{i}$ and $v_{j}$ meet in a memory cycle T
$RT_{i}$	Relation Tree of node $v_{i}$
$L_{i}\left(m_{a}\right)$	Number of copies of $m_{a}$ carried by node $v_{i}$
$\beta_{now}$	Current cache occupancy of node
$\beta_{best}$	Optimal cache occupancy of node
L	The actual number of copies
$L_{init}$	The initial number of copies
$TTL_{new}$	The lifetime of the newly generated message copy
$TTL_{old}$	The original lifetime of the message copy

**Table 2:** Scenario parameters.

Parameter	Default Value	Variation Range
Scenario Size (m^2^)	4500 × 3400	-
Simulation Time (h)	10	-
Message Generation Interval (s)	30	10–50
Message Size (KB)	1250	1000–1500
Buffer Size (MB)	15	5–25
TTL (min)	60	20–100
Number of Nodes	300	100–500
$L_{init}$	8	-
$\beta_{best}$	0.6	-
∂	2	-
M	3	-
T (min)	30	-

**Table 3:** Node attributes.

Group Number	Parameter	Value
Group 1 (Pedestrians)	movement speed (m/s)	0.5–1.5
	communication rate (Kbps)	250
	communication range (m)	30
Group 2 (Bus)	movement speed (m/s)	2.7–13.9
	communication rate (Kbps)	250
	communication range (m)	30
Group 3 (Taxi)	movement speed (m/s)	3–12
	communication rate (Kbps)	250
	communication range (m)	30
Group 4 (Tram)	movement speed (m/s)	7–10
	communication rate (Mbps)	10
	communication range (m)	150

## Data Availability

Not applicable.
